# Supplementary material for: Fluorescent protein lifetimes report densities and phases of nuclear condensates during embryonic stem-cell differentiation
Source: Nat Commun. 2023 Aug 12;14:4885. doi: 10.1038/s41467-023-40647-6 (PMC10423231; doi:10.1038/s41467-023-40647-6)
Supplement: Supplementary file 1 — Supplementary Information [file 41467_2023_40647_MOESM1_ESM.pdf]

Supplementary Information

Fluorescent protein lifetimes report increased local densities and phases of nuclear condensates during embryonic stem cell differentiation

Khalil Joron[1,*], Juliane Oliveira Viegas[2,*], Liam Haas-Neill[3], Sariel Bier[1,2], Paz Drori[1], Shani Dvir[1], Patrick Siang Lin Lim[2], Sarah Rauscher[3], Eran Meshorer[2,4,‡], Eitan Lerner[1,5,‡]

[1] Department of Biological Chemistry, The Alexander Silberman Institute of Life Sciences, Faculty of Mathematics & Science, The Edmond J. Safra Campus, The Hebrew University of Jerusalem, Jerusalem 9190401, Israel.

[2] Department of Genetics, The Alexander Silberman Institute of Life Sciences, The Hebrew University of Jerusalem, Edmond J. Safra Campus, Givat Ram, Jerusalem, 91904, Israel.

[3] Department of Chemical and Physical Sciences, University of Toronto Mississauga, Mississauga, ON, L5L 1C6, Canada; Department of Physics, University of Toronto, Toronto, ON, M5S 1A7, Canada; Department of Chemistry, University of Toronto, 80 St. George Street, Toronto, ON M5S 3H6, Canada.

[4] Edmond and Lily Center for Brain Sciences (ELSC), The Hebrew University of Jerusalem, Jerusalem, 9190401, Israel.

[5] The Center for Nanoscience and Nanotechnology, The Hebrew University of Jerusalem, Jerusalem 9190401, Israel.

[*] Shared first authors

[‡] Corresponding authors

Corresponding authors: eran.meshorer@mail.huji.ac.il; eitan.lerner@mail.huji.ac.il

Supplementary Information and Equations

Retrieving the fluorescence lifetime values from fitting models to fluorescence decays

Theoretical models fitted to fluorescence decays (Eq. 1):

$$I(t) \propto \mathrm{irf}(t) \otimes \left(\alpha_1 e^{-\frac{t}{\tau_1}} + \alpha_2 e^{-\frac{t}{\tau_2}} \right) \quad (1)$$

where t is photon nanotime, the time between the moment of excitation and the moment of photon detection, $I(t)$ is the photon counts as a function of photon nanotime, $\mathrm{irf}(t)$ is the impulse response function (IRF) of the system, and τ_1, τ_2, and α_1, α_2, are the two lifetime components and their amplitudes, respectively. The ⊗ operator is a commonly used symbol of the convolution operation. Then, the two lifetime components are used for the calculation of the intrinsic average lifetime, $\bar{\tau}$ (Eq. 2):

$$\bar{\tau} = \frac{\alpha_1 \tau_1^2 + \alpha_2 \tau_2^2}{\alpha_1 \tau_1 + \alpha_2 \tau_2} \quad (2)$$

Retrieving the fluorescence anisotropy decay curves from polarized fluorescence decays

The fluorescence anisotropy decay, $r(t)$, was calculated from the polarized fluorescence decays as follows (Eq. 3):

$$r(t) = \frac{I_{\parallel}(t) - G \cdot I_{\perp}(t)}{I_{\parallel}(t) + 2G \cdot I_{\perp}(t)} \quad (3)$$

where $I_{\parallel}(t)$ and $I_{\perp}(t)$ are the decays of fluorescence polarized parallel- and perpendicular relative to the excitation polarization, t is the time following the moment of excitation, and G is the well-known G-factor which represents deviations from equality of $I_{\parallel}$ and $I_{\perp}$ in samples not exhibiting fluorescence anisotropy.

The fluorescence anisotropy due to a given depolarization mode, r, depends both on the fluorescence lifetime, τ, and the rotational correlation time, θ, following the Perrin equation (Eq. 4):

$$\frac{r_0}{r} = 1 + \frac{\tau}{\theta} \quad (4)$$

Where, r_0, the fundamental anisotropy, is a constant for a given dye system. Importantly, the rotational correlation time is influenced either by temperature, viscosity or size of the rotating system, following the Stokes-Einstein relationship, or due to faster depolarization that can be caused by energy transfer.

In-cell FCS calculations

The fluorescence autocorrelation curves, $G(\tau)$, were calculated from the acquired fluorescence intensity trajectories, $I(t)$ (Eq. 5):

$$G(\tau) = \frac{\langle \delta I(t) \cdot \delta I(t+\tau) \rangle}{\langle I(t) \rangle^2} \quad (5)$$

where t is the absolute photon arrival time, τ is the time lag between a pair of photon arrival times, δ denotes the deviation relative to the mean value and $\langle \, \rangle$ denote a time average.

Then, the resulting fluorescence autocorrelation curves were fitted to a model that takes into account fluorescence fluctuations due to the diffusion of two species in and out of the laser focus, fast and slower diffusing species (Eq. 6):

$$G(\tau) = \frac{1}{\bar{N}} \cdot \left\{ \frac{f}{\left(1+\frac{\tau}{\tau_{D,fast}}\right)\sqrt{1+\left(\frac{\omega_0}{z_0}\right)^2 \cdot \frac{\tau}{\tau_{D,fast}}}} + \frac{1-f}{\left(1+\frac{\tau}{\tau_{D,slow}}\right)\sqrt{1+\left(\frac{\omega_0}{z_0}\right)^2 \cdot \frac{\tau}{\tau_{D,slow}}}} \right\} \quad (6)$$

where $\bar{N}$ is the mean number of molecules in the laser focus, f is the fraction of the fast species, $\tau_{D,fast}$ and $\tau_{D,slow}$ are the diffusion times through the laser focus of the fast and slow diffusing species, respectively, and ω_0 and z_0 are the axial and longitudal lengths of the laser focus. Fitting was performed after constraining the model parameters so that all parameters should have positive values, the fraction parameter should not exceed a value of 1 and the ratio of the longitudinal and axial lengths of the laser focus should be bound within the typical 4 – 10 range of values.

Analyses of photobleaching rates

The acquired in-cell fluorescence trajectories are exhibiting two timescales of photobleaching, slow and fast, representing rapidly and slowly diffusing species. Therefore, each in-cell fluorescence intensity trajectory was fitted with a bi-exponential decay model (Eq. 7):

$$I(t) = a_{fast} e^{-\frac{t}{t_{fast}}} + a_{slow} e^{-\frac{t}{t_{slow}}} + y_0 \quad (7)$$

where t_{fast} and t_{slow} are the lifetimes of the fast and slow photobleaching process, a_{fast} and a_{slow} are their amplitude and y_0 is a baseline representing species that diffuse fast enough that they do not photobleach even within the 2 minutes of acquisition.

Supplementary Figures

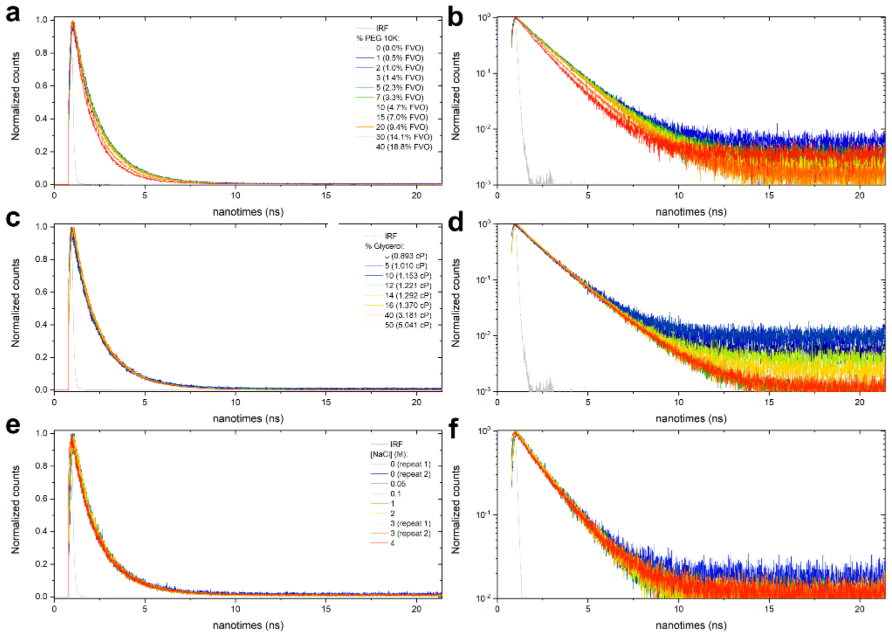

Supplementary Fig. 1: mCherry normalized fluorescence decays at different conditions. (a, c, e) linear scale. **(b, d, f**) log-scale. **(a, b)**, fluorescence decays of 100 nM mCherry as a function of PEG 10,000; **(c, d)** decays as a function of glycerol; **(e, f)** decays as a function of NaCl.

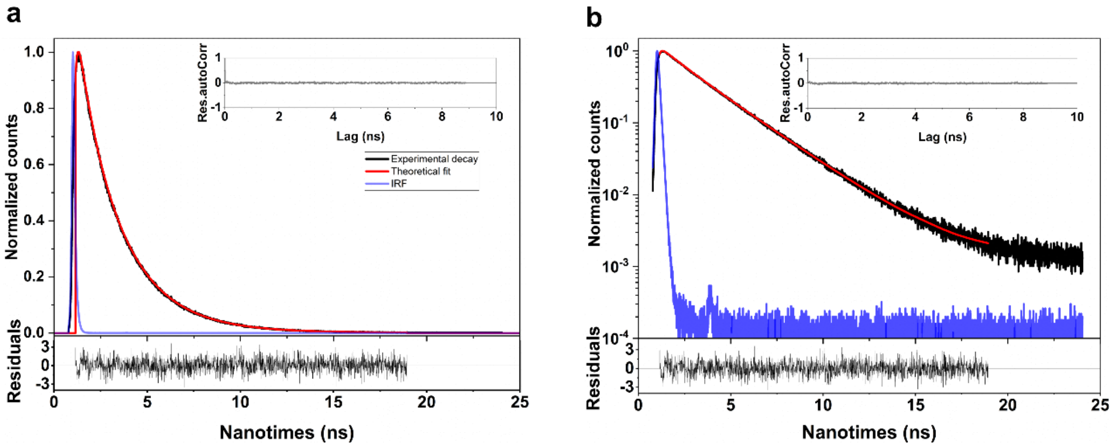

Supplementary Fig. 2: An example of the fluorescence decay fitting procedure. The fluorescence decay fitting is shown in linear **a**, and log scales **b**, respectively. Experimental decay (black), the IRF (blue) and the best-fit model (red) are shown. The fitting residuals (bottom panels) and their auto-correlation (panel insets) are also shown. This example is the best-fit of the fluorescence decay of eGFP in the presence of 1.3 M trehalose, with reduced χ^2 of 1.029.

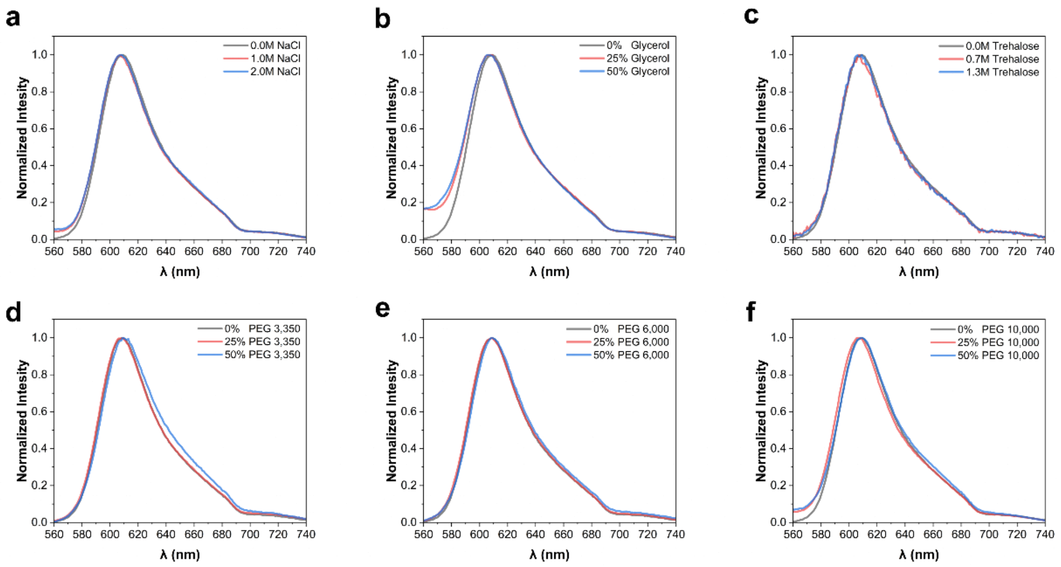

Supplementary Fig. 3: mCherry normalized fluorescence spectra at different conditions. The fluorescence spectra of 100 nM mCherry were measured at different concentrations of different conditions: **a**, NaCl, **b**, Glycerol, **c**, Trehalose, **d**, PEG 3,350, **e**, PEG 6,000 and **f**, PEG 10,000.

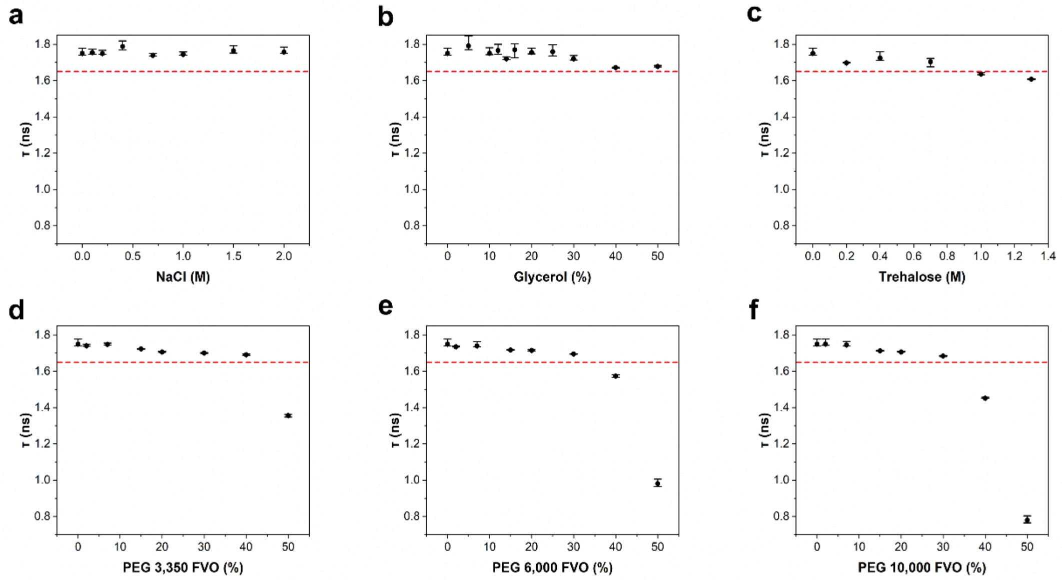

Supplementary Fig. 4: The mean fluorescence lifetime of mRFP at different physico-chemical conditions. mean fluorescence lifetime (τ) of 100 nM mRFP as a measure of increasing NaCl concentrations **a**, Glycerol, **b**, Trehalose, **c**, and **d-f**, PEG. Crowding exclusively induces lifetime reduction, and only above 35-45% FVO. The values and error estimates are based on calculations (Eq. S2) using best-fit values of the recorded fluorescence decays (Fig. S1) to a sum of two exponents function (Eq. S1). The values of the intrinsic mean fluorescence lifetimes are reported in Table S1. The error estimates were calculated directly from the fitting uncertainty, and are reported as the minimal and maximal intrinsic mean fluorescence lifetime values calculated from all lifetime component values and their amplitudes, using Supplementary Equation 2, which have a reduced χ^2 values within 95% confidence relative to the best-fit minimal reduced χ^2 value. Red line that is set at 1.65 ns highlights the limit below which fluorescence lifetimes drop in the presence of elevated concentrations of PEG or trehalose. Titration of NaCl or glycerol, leads to non-monotonic changes in τ which fluctuate around the typical fluorescence lifetime of mRFP.

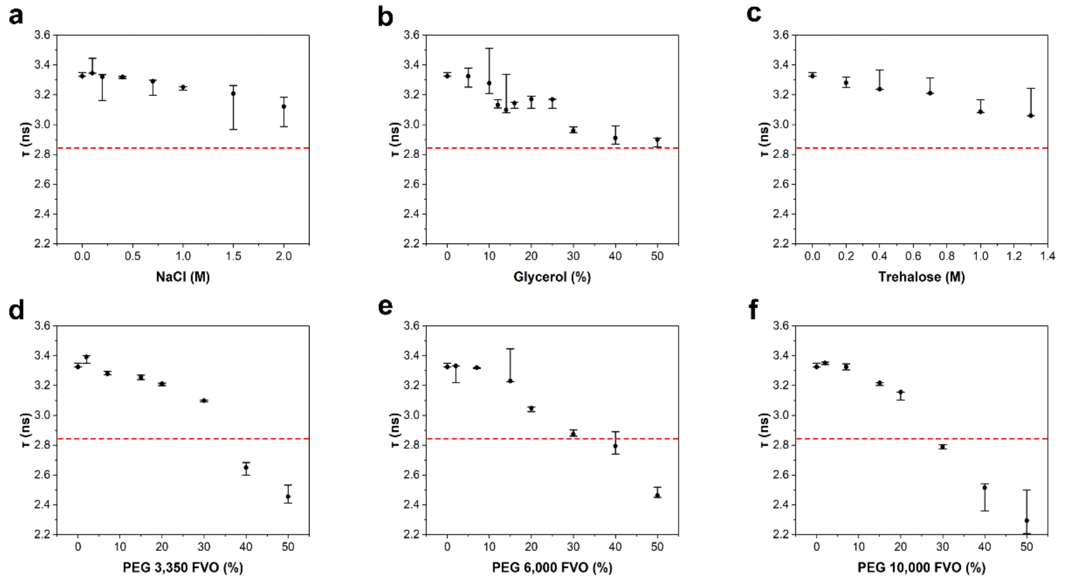

Supplementary Fig. 5: The mean fluorescence lifetime of mCitrine at different physico-chemical conditions. mean fluorescence lifetime (τ) of 100 nM mCitrine as a measure of increasing NaCl concentrations **a**, Glycerol, **b**, Trehalose, **c**, and **d-f**, PEG. Crowding exclusively induces lifetime reduction, and only above 30% FVO. The values and error estimates are based on calculations (Eq. S2) using best-fit values of the recorded fluorescence decays (Fig. S1) to a sum of two exponents function (Eq. S1). The values of the intrinsic mean fluorescence lifetimes are reported in Table S1. The error estimates were calculated directly from the fitting uncertainty, and are reported as the minimal and maximal intrinsic mean fluorescence lifetime values calculated from all lifetime component values and their amplitudes, using Supplementary Equation 2, which have a reduced χ^2 values within 95% confidence relative to the best-fit minimal reduced χ^2 value. Red line that is set at 2.84 ns highlights the limit below which fluorescence lifetimes drop in the presence of elevated concentrations of PEG or trehalose.

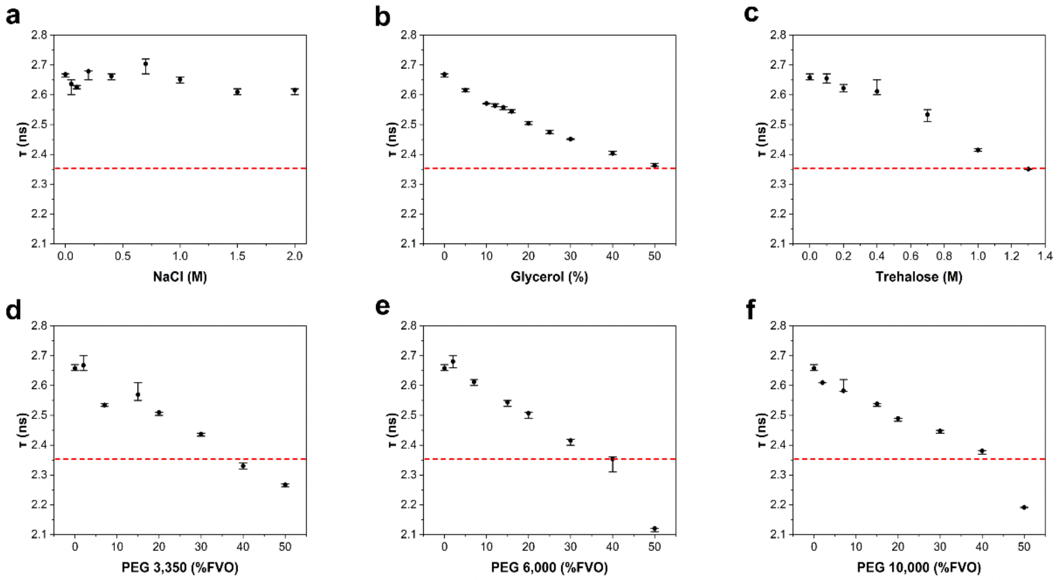

Supplementary Fig. 6: The mean fluorescence lifetime values of eGFP at different concentrations of different physico-chemical conditions. mean fluorescence lifetime (τ) of 100 nM eGFP as a measure of increasing NaCl concentrations **a**, Glycerol, **b**, Trehalose, **c**, and **d-f**, PEG. Viscosity and crowding induce lifetime reduction. Fluorescence lifetime reduction from the typical 2.5-2.7 ns is induced above 40% FVO. The values of the intrinsic mean fluorescence lifetimes are reported in Table S1. The values of the intrinsic mean fluorescence lifetimes are reported in Table S1. The error estimates were calculated directly from the fitting uncertainty, and are reported as the minimal and maximal intrinsic mean fluorescence lifetime values calculated from all lifetime component values and their amplitudes, using Supplementary Equation 2, which have a reduced χ^2 values within 95% confidence relative to the best-fit minimal reduced χ^2 value. Red line that is set at 2.35 ns highlights the limit below which fluorescence lifetimes drop in the presence of elevated concentrations of PEG or trehalose.

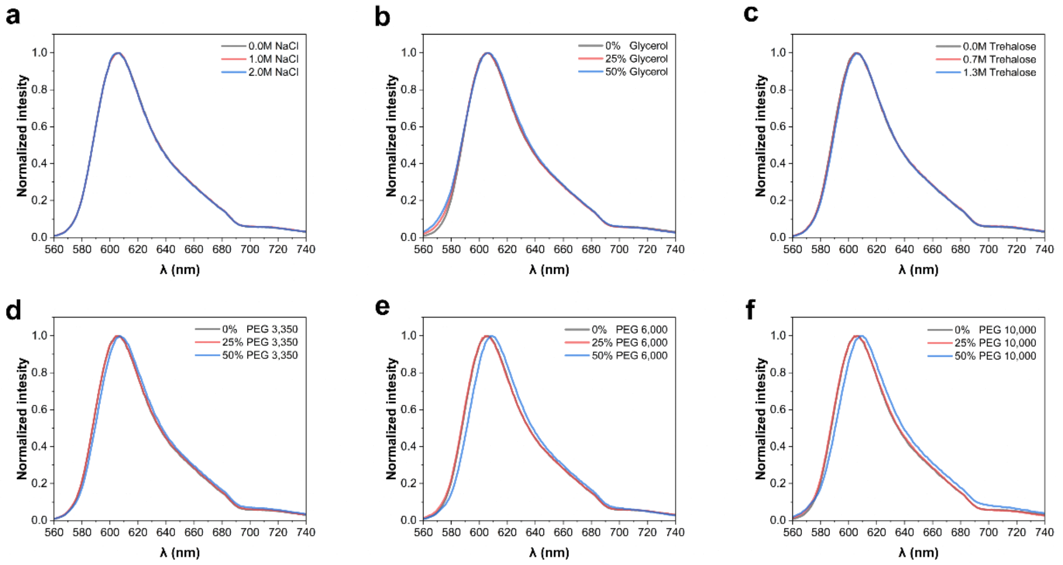

Supplementary Fig. 7: mRFP normalized fluorescence spectra at different conditions. The fluorescence spectra of 100 nM mRFP were measured at different concentrations of different conditions: **a**, NaCl, **b**, Glycerol, **c**, Trehalose, **d**, PEG 3,350, **e**, PEG 6,000 and **f**, PEG 10,000.

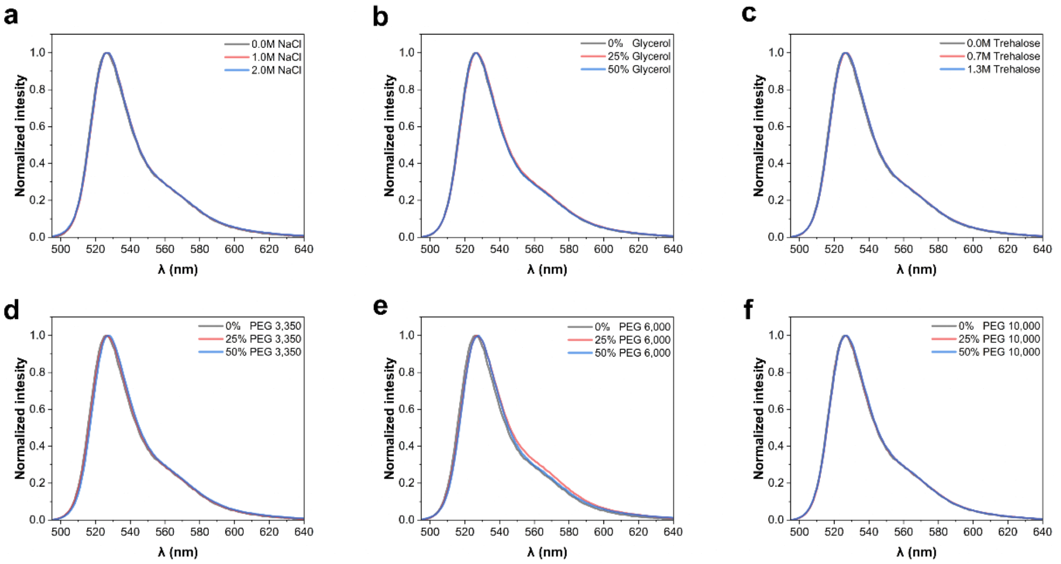

Supplementary Fig. 8: mCitrine normalized fluorescence spectra at different conditions. The fluorescence spectra of 100 nM mCitrine were measured at different concentrations of different conditions: **a**, NaCl, **b**, Glycerol, **c**, Trehalose, **d**, PEG 3,350, **e**, PEG 6,000 and **f**, PEG 10,000.

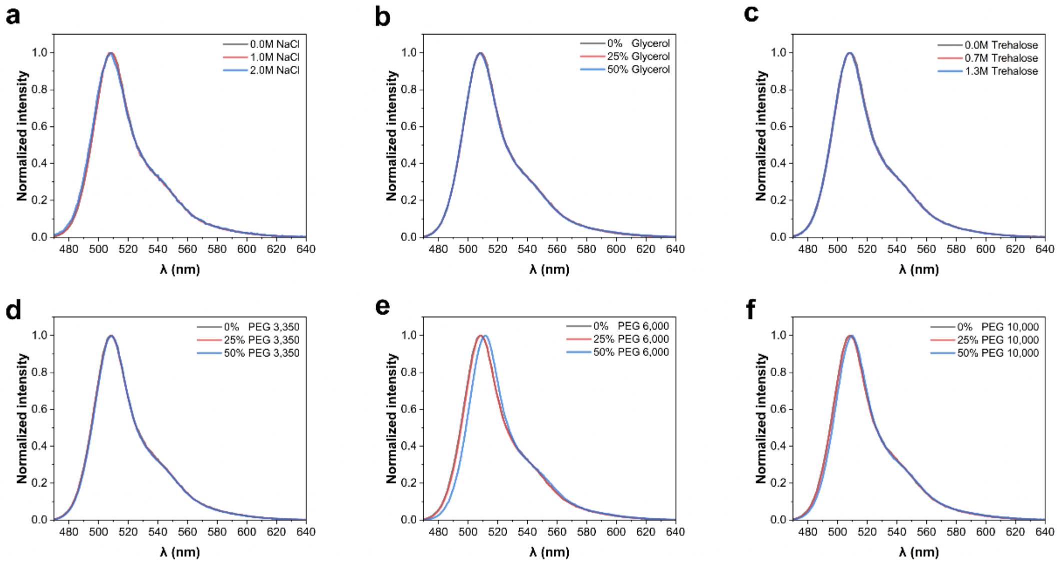

Supplementary Fig. 9: eGFP normalized fluorescence spectra at different conditions. The fluorescence spectra of 100 nM eGFP were measured at different concentrations of different conditions: **a**, NaCl, **b**, Glycerol, **c**, Trehalose, **d**, PEG 3,350, **e**, PEG 6,000 and **f**, PEG 10,000.

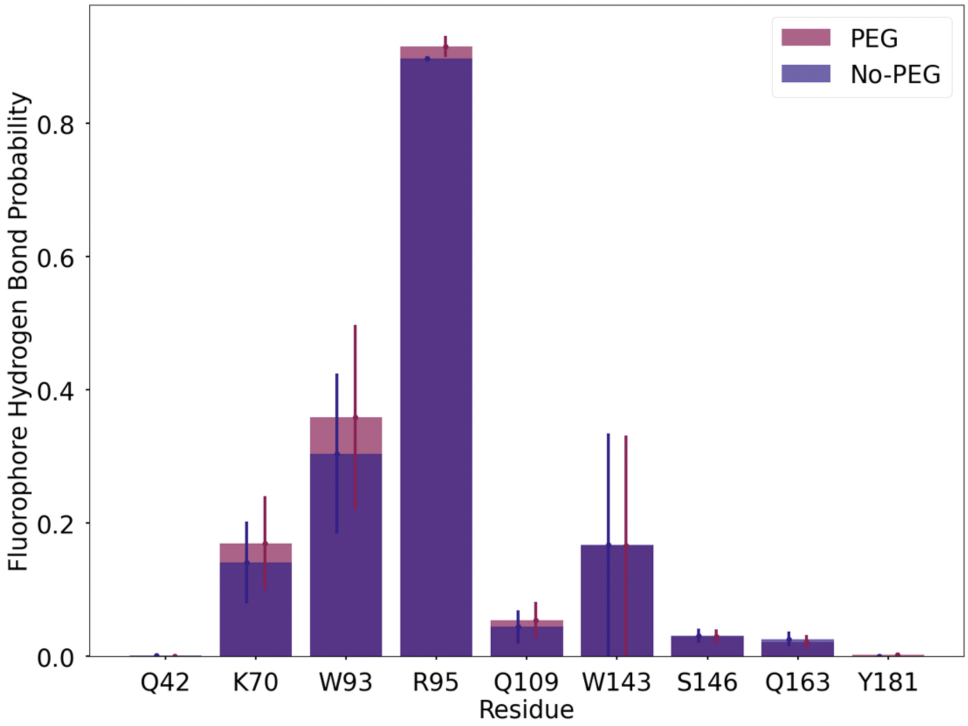

Supplementary Fig. 10: Protein-fluorophore hydrogen bonds. The probability of hydrogen bond formation between the fluorophore and other protein residues for each system. Values for the PEG system are shown in plum and values for the no-PEG system are shown in navy blue. Uncertainty is shown as error bars, computed as the SEM treating each trajectory as an independent measurement.

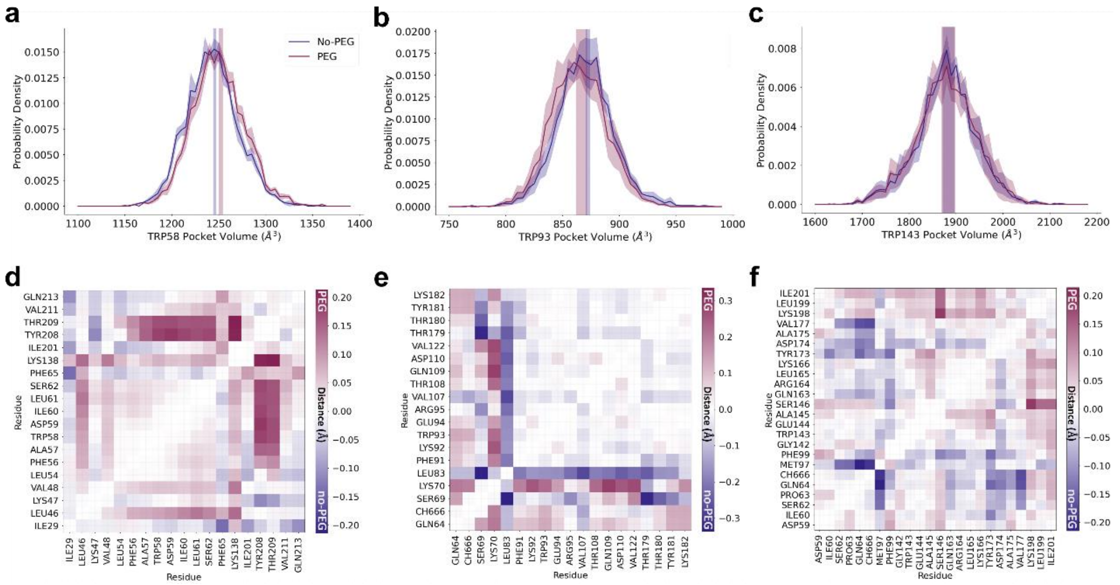

Supplementary Fig. 11: Trp environments. **a-c** show the distributions of Trp pocket volumes for Trp's 58, 93, and 143, respectively. The distributions for the PEG systems are shown in plum, and the distributions for the systems without PEG are shown in navy. Shaded regions indicate the standard error of the mean, treating each trajectory as an independent observation. **d-f** show the difference map of the average Trp-pocket C_α-C_α distances for Trp's 58, 93, and 143, respectively. Distances that are larger in the PEG system are positive and shown in plum, and ones that are larger in the no-PEG system are negative and shown in navy.

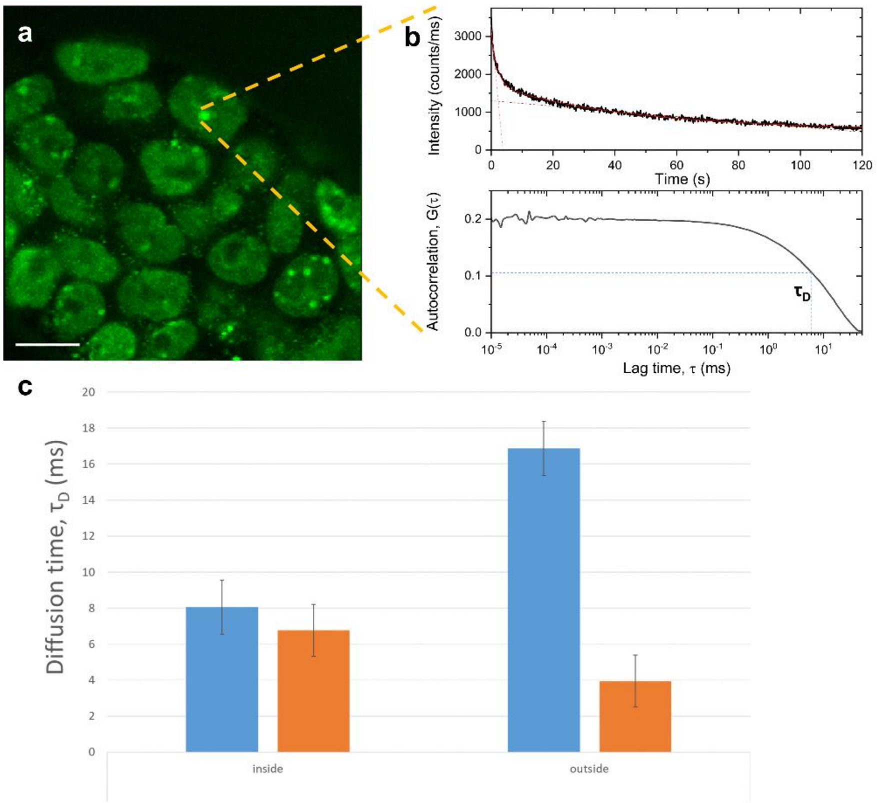

Supplementary Fig. 12: In-cell FCS report on diffusivities within HP1α condensates. a, HP1α-mCherry image shows nuclei with bright foci. Scale bar 10 µm. We position the laser focus onto a given position within one such HP1α-mCherry condensate (yellow dashed arrows), and acquire fluorescence for a given acquisition time (e.g., 2 minutes). **b,** The fluorescence trajectory acquired (top) from the chosen position within the HP1α-mCherry condensate exhibits a monotonic decrease due to photobleaching. This intensity trajectory is fitted to a bi-exponential decay to characterize the photobleaching rates. The fluorescence trajectory is noisy, and this noise carries information regarding the temporal fluctuations in the number of molecules within the laser focus and the diffusion times of the mCherry-tagged HP1α molecules traversing the laser focus. The fluctuations and diffusion times are retrieved from the autocorrelation (bottom panel) of the fluorescence trajectory. The characteristic diffusion time, τ_D (blue dashed line), is retrieved by model fitting (Eq. 4) to the fluorescence autocorrelation curve (Eq. S3). Of note is that the timescales covered by the fluorescence autocorrelation curve (bottom; <100 ms) are faster than the two photobleaching decay times observed in the acquired fluorescence trajectory (top; seconds and tens of seconds). **c,** Averages of diffusion times inside and outside multiple HP1α-mCherry condensates before (blue; inside, n=18; outside, n=10) and after (orange; inside, n=10, outside, n=5) early differentiation are reported. Despite the reported differences in diffusion times, the general conclusion is that diffusion of HP1α-mCherry occur within condensates under all conditions tested, and hence a solid phase can be ruled-out. Data in **c** are represented as best-fit values ± SD error estimates from model fitting.

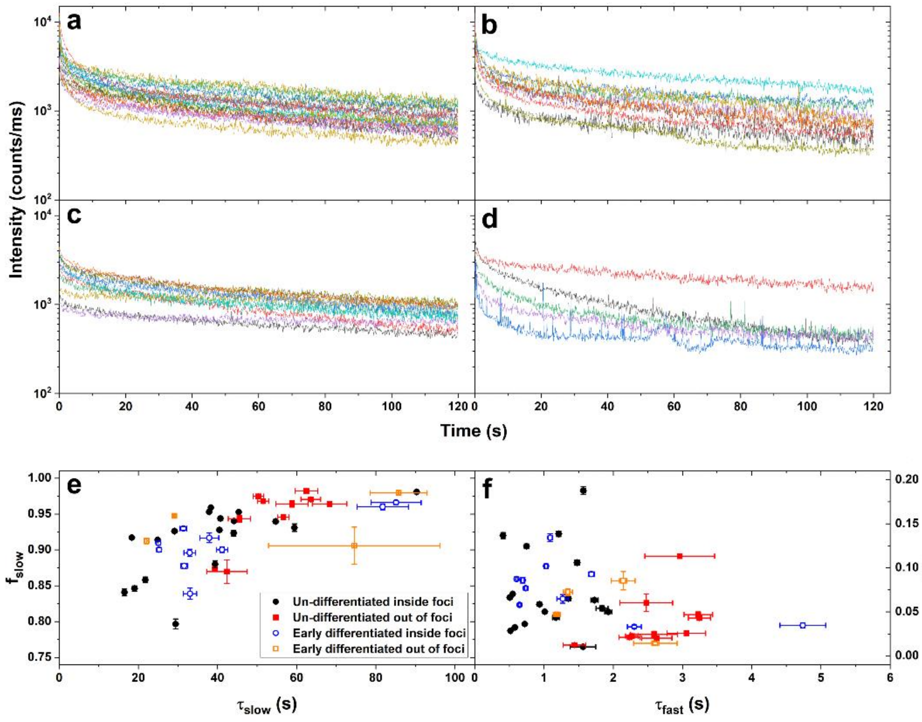

Supplementary Fig. 13: Photobleaching rates analyses of in-cell fluorescence intensity trajectories. a-d, Multiple 2-minute fluorescence intensity trajectories were recorded inside (**a**, **b**) and outside (**c**, **d**) of HP1α-mCherry foci, in un-differentiated (**a**, **c**) and early differentiated (**b**, **d**) ESCs. **e, f**, summarizes the fractions of the slow and fast photobleaching processes, and their times inside HP1α-mCherry foci and out-of-foci in un-differentiated and in early differentiated ESCs. Data are presented as best-fit values ± SD error estimates from model-fitting per each best-fit parameter of each point in the cells in which photobleaching data were acquired and analyzed.

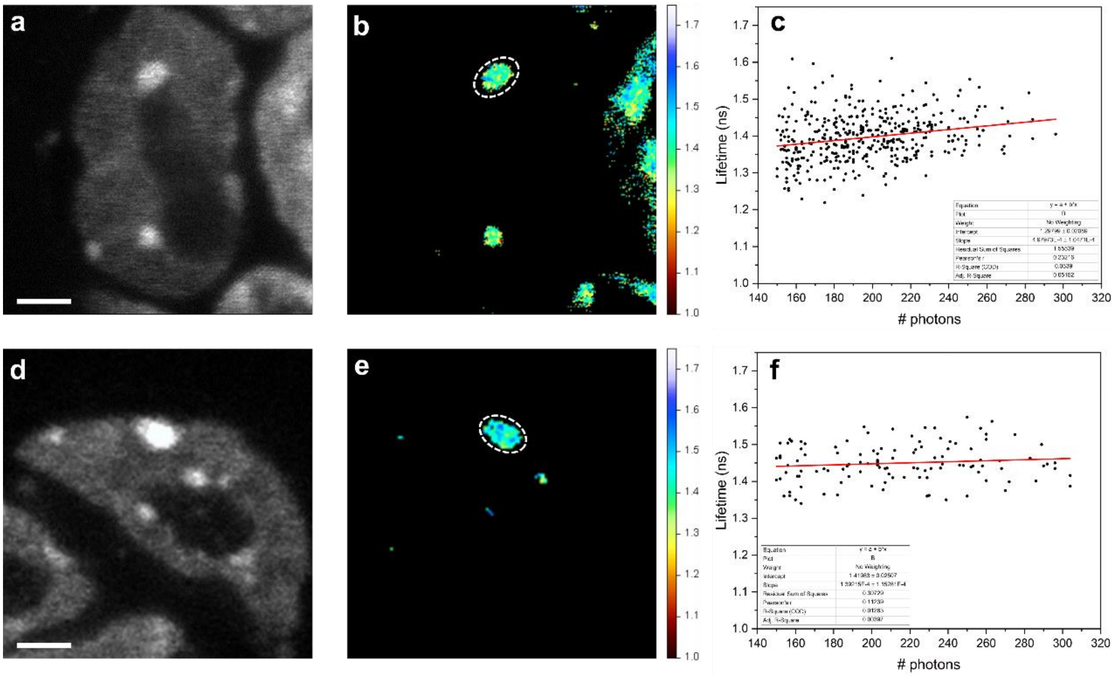

Supplementary Fig. 14: Fluorescence intensities and lifetimes within HP1α condensates exhibit at most weak correlation. a-f, Two examples of undifferentiated ESCs showing HP1α condensates (**a**, **b** and **d**, **e**) with heterogeneity in fluorescence lifetimes (**b**, **e**). The pearson correlation coefficient values between pixel intensity (**a**, **d**) and lifetime (**b**, **e**) are 0.23 (**c**) and 0.11 (**f**), pointing towards a weak correlation at most between changes in intensity and changes in lifetimes (**c**, **f**). Scale bar 2 µm. Fluorescence lifetime colorbars scale from 1.00 and 1.75 ns.

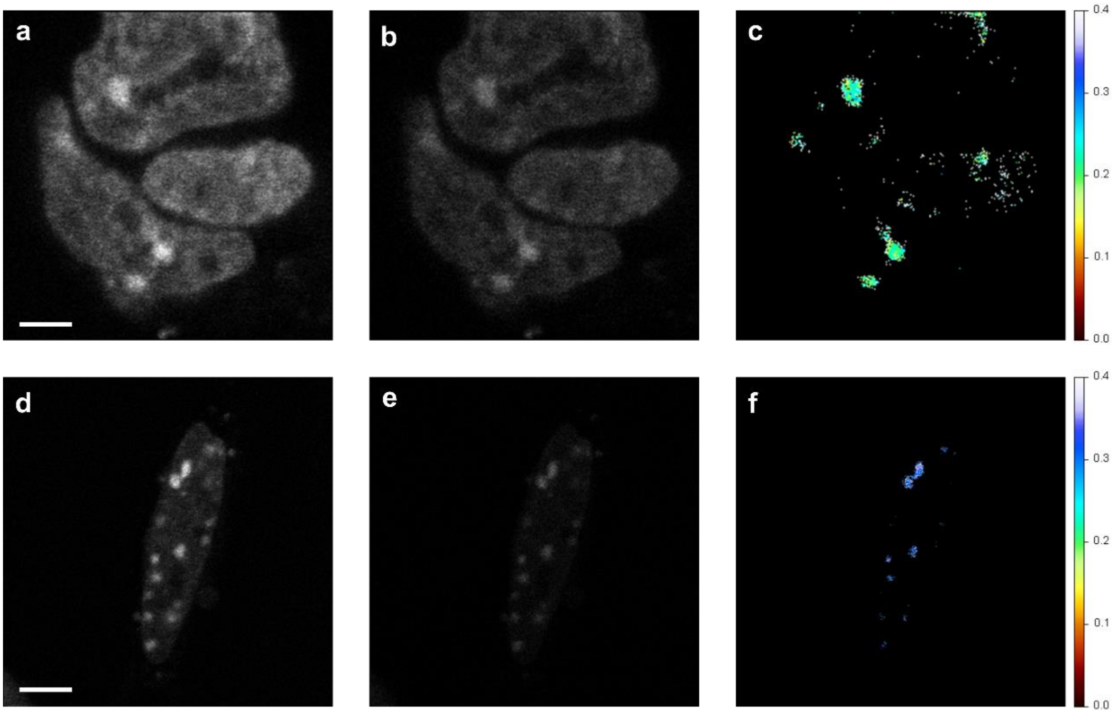

Supplementary Fig. 15: Fluorescence anisotropy images recover differences between HP1α condensates in undifferentiated and early-differentiated ESCs. a, **b**, **d**, **e**, fluorescence intensity images of fluorescence polarized parallel (**a**, **d**) and perpendicular (**b**, **e**) relative to the polarization of excitation, in undifferentiated (**a**, **b**) and early-differentiated (**d**, **e**) cells. **c**, **f,** the calculated fluorescence anisotropy images for pixels within HP1α condensates, in undifferentiated (**c**) and early-differentiated (**f**) cells. Scale bar 2 µm.

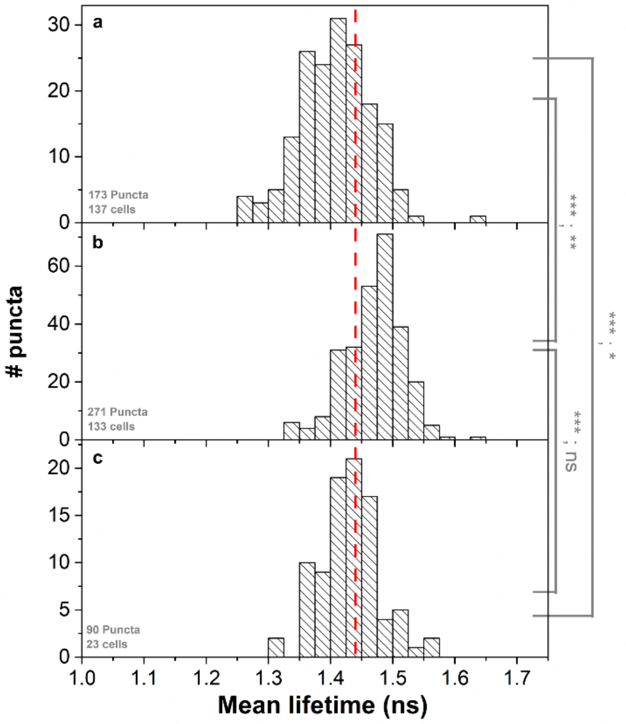

Supplementary Fig. 16: Mean fluorescence lifetimes of mCherry-HP1α condensates from undifferentiated ESCs, early-differentiated ESCs and ESCs transfected with LNA probes. **a,** Undifferentiated ESCs; **b,** Early-differentiated ESCs and **c,** Undifferentiated ESCs transfected with LNA probes. Histograms show the distribution of mean fluorescence lifetimes of different mCherry-HP1α puncta. Asterisks report on the p-value from two-sided T-test and from two-sided F-test, comparing means and variances of histograms. p>0.05 n.s.; 0.01<p<0.05*; 0.001<p<0.01**; p<0.001***. A vertical red line shows the 1.44 ns lower boundary of mCherry fluorescence lifetime below which the crowdedness level is above 30% FVO (Fig. 1).

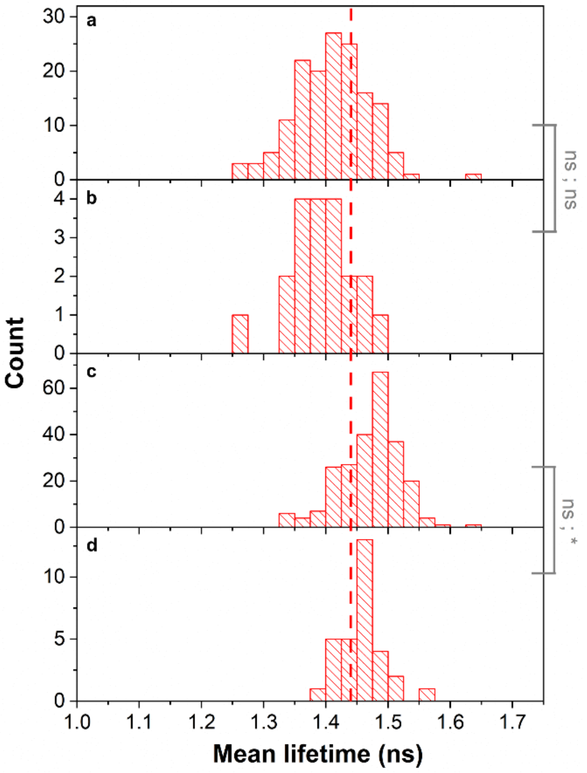

Supplementary Fig. 17: Mean fluorescence lifetimes in mCherry-HP1α condensates from undifferentiated and early-differentiated ESCs in the absence and presence of transfected RNA scramble probe. **a**, **b**, A comparison of the mean fluorescence lifetime histograms in undifferentiated ESCs in the absence (**a**) and presence (**b**) of transfected RNA scramble probe, showing lack of significant differences, allowing their summation. **c**, **d**, A comparison of the mean fluorescence lifetime histograms in early-differentiated ESCs in the absence (**c**) and presence (**d**) of transfected RNA scramble probe, showing lack of significant differences, allowing their summation. Asterisks report on the p-value from two-sided T-test and from two-sided F-test, comparing means and variances of histograms. $p>0.05$ n.s.; $0.01<p<0.05^{*}$; $0.001<p<0.01^{**}$; $p<0.001^{***}$. A vertical red line shows the 1.44 ns lower boundary of mCherry fluorescence lifetime below which the crowdedness level is above 30% FVO (Fig. 1).

Supplementary Tables

Supplementary Table 1: best-fit results of fluorescence decay fittings and intrinsic average lifetimes. The error estimates are the minimal and maximal intrinsic mean fluorescence lifetime values calculated from all lifetime component values and their amplitudes, which are within a reduced χ^2 that is within 95% confidence relative to the minimal best-fit reduced χ^2 value.

FP	Additive	Concentration (M)/Viscosity (% glycerol)/Crowding (%FVO)	α_1	τ_1 (ns)	α_2	τ_2 (ns)	$\bar{\tau}$ (ns)	χ_R^2
mCherry	NaCl	0.0 (M)	4.33	1.59	1.59	0.83	1.46 (1.46-1.47)	1.240
mCherry	NaCl	0.05 (M)	5.70	1.55	1.48	0.74	1.47 (1.46-1.47)	1.216
mCherry	NaCl	0.1 (M)	5.94	1.45	0.35	1.45	1.45 (1.44-1.46)	1.085
mCherry	NaCl	0.2 (M)	5.94	1.50	1.04	0.48	1.45 (1.44-1.45)	1.077
mCherry	NaCl	0.4 (M)	5.72	1.50	1.00	0.50	1.44 (1.44-1.45)	1.135
mCherry	NaCl	0.7 (M)	5.90	1.50	1.00	0.52	1.45 (1.44-1.46)	1.045
mCherry	NaCl	1.0 (M)	1.97	1.18	0.95	1.91	1.50 (1.49-1.52)	1.092
mCherry	NaCl	1.5 (M)	0.91	0.82	2.43	1.59	1.47 (1.46-1.48)	1.113
mCherry	NaCl	2.0 (M)	1.16	0.95	1.99	1.66	1.48 (1.47-1.49)	1.174
mCherry	Trehalose	0.0 (M)	2.35	1.17	1.41	1.87	1.52 (1.50-1.53)	1.275
mCherry	Trehalose	0.2 (M)	0.71	0.78	2.47	1.59	1.49 (1.48-1.50)	1.179
mCherry	Trehalose	0.4 (M)	0.81	0.78	2.89	1.58	1.49 (1.48-1.49)	1.196
mCherry	Trehalose	0.7 (M)	3.93	1.53	0.73	0.56	1.46 (1.45-1.47)	1.165
mCherry	Trehalose	1.0 (M)	0.83	0.43	4.69	1.48	1.43 (1.42-1.43)	1.110
mCherry	Trehalose	1.3 (M)	6.58	1.39	1.47	0.34	1.34 (1.33-1.35)	1.091
mCherry	Glycerol	0 (%)	6.31	1.47	-	-	1.47 (1.45-1.48)	1.158
mCherry	Glycerol	5 (%)	3.50	1.58	0.82	0.76	1.50 (1.48-1.51)	1.152
mCherry	Glycerol	10 (%)	4.23	1.53	0.67	0.54	1.48 (1.47-1.49)	1.075
mCherry	Glycerol	12 (%)	1.01	0.98	2.24	1.65	1.51 (1.49-1.52)	1.129
mCherry	Glycerol	14 (%)	7.31	1.54	1.14	0.48	1.49 (1.48-1.49)	1.093
mCherry	Glycerol	16 (%)	4.11	1.56	0.84	0.66	1.49 (1.48-1.50)	1.127
mCherry	Glycerol	20 (%)	1.05	0.35	7.53	1.51	1.47 (1.46-1.48)	1.103
mCherry	Glycerol	25 (%)	4.47	1.31	3.54	1.67	1.49 (1.47-1.49)	1.116
mCherry	Glycerol	30 (%)	8.34	1.52	0.87	0.47	1.48 (1.48-1.49)	1.101
mCherry	Glycerol	40 (%)	3.66	1.68	6.05	1.36	1.49 (1.48-1.49)	1.108
mCherry	Glycerol	50 (%)	5.83	1.57	1.22	0.54	1.50 (1.49-1.51)	1.102
mCherry	PEG 3,350	0 (% FVO)	0.99	0.66	6.62	1.55	1.50 (1.49-1.50)	1.586
mCherry	PEG 3,350	2 (% FVO)	5.08	1.52	0.66	0.54	1.48 (1.47-1.49)	1.204
mCherry	PEG 3,350	7 (% FVO)	5.90	1.57	1.50	0.76	1.48 (1.48-1.49)	1.161
mCherry	PEG 3,350	15 (% FVO)	9.58	1.50	1.45	0.36	1.46 (1.45-1.47)	1.168
mCherry	PEG 3,350	20 (% FVO)	12.40	1.49	1.87	0.33	1.45 (1.44-1.46)	1.219
mCherry	PEG 3,350	30 (% FVO)	11.72	1.50	2.27	0.52	1.44 (1.43-1.45)	1.125
mCherry	PEG 3,350	40 (% FVO)	8.87	1.38	2.07	0.44	1.32 (1.31-1.32)	1.178
mCherry	PEG 3,350	50 (% FVO)	6.55	1.39	2.09	0.54	1.30 (1.29-1.30)	1.184
mCherry	PEG 6,000	0 (% FVO)	4.34	1.59	1.59	0.83	1.46 (1.46-1.47)	1.240
mCherry	PEG 6,000	2 (% FVO)	5.81	1.51	0.96	0.52	1.46 (1.45-1.46)	1.136
mCherry	PEG 6,000	7 (% FVO)	5.74	1.50	0.99	0.52	1.45 (1.44-1.45)	1.137
mCherry	PEG 6,000	15 (% FVO)	8.31	1.49	1.78	0.46	1.44 (1.43-1.45)	1.201
mCherry	PEG 6,000	20 (% FVO)	8.45	1.49	1.57	0.44	1.44 (1.43-1.45)	1.223
mCherry	PEG 6,000	30 (% FVO)	8.90	1.50	1.82	0.52	1.44 (1.43-1.45)	1.210
mCherry	PEG 6,000	40 (% FVO)	4.49	1.49	2.17	0.66	1.35 (1.34-1.35)	1.158
mCherry	PEG 6,000	50 (% FVO)	4.86	1.27	1.95	0.54	1.17 (1.16-1.17)	1.203
mCherry	PEG 10,000	0 (% FVO)	4.34	1.59	1.59	0.83	1.46 (1.46-1.47)	1.298
mCherry	PEG 10,000	2 (% FVO)	6.01	1.51	0.98	0.49	1.46 (1.45-1.46)	1.110
mCherry	PEG 10,000	7 (% FVO)	8.29	1.49	1.12	0.40	1.45 (1.44-1.46)	1.147
mCherry	PEG 10,000	15 (% FVO)	7.59	1.50	1.28	0.46	1.45 (1.44-1.45)	1.194
mCherry	PEG 10,000	20 (% FVO)	9.46	1.50	1.78	0.46	1.44 (1.43-1.45)	1.161
mCherry	PEG 10,000	30 (% FVO)	6.97	1.50	2.05	0.55	1.41 (1.40-1.42)	1.176
mCherry	PEG 10,000	40 (% FVO)	6.14	1.45	4.13	0.61	1.27 (1.26-1.27)	1.153
mCherry	PEG 10,000	50 (% FVO)	2.66	0.92	1.53	1.61	1.26 (1.25-1.27)	1.192
mRFP	NaCl	0.0 (M)	3.21	1.50	0.97	2.29	1.75(1.74-1.77)	1.080
mRFP	NaCl	0.1 (M)	0.96	1.29	1.74	1.93	1.75(1.74-1.77)	1.045
mRFP	NaCl	0.2 (M)	2.21	1.87	0.83	1.27	1.75(1.74-1.76)	1.072
mRFP	NaCl	0.4 (M)	2.20	1.62	0.19	2.83	1.78(1.76-1.81)	1.130
mRFP	NaCl	0.7 (M)	3.02	1.83	0.76	1.15	1.73(1.73-1.74)	1.055
mRFP	NaCl	1.0 (M)	0.76	1.15	2.62	1.85	1.74(1.73-1.75)	1.046
mRFP	NaCl	1.5 (M)	2.14	1.54	0.83	2.18	1.76(1.75-1.79)	1.108

	mRFP	NaCl	2.0 (M)	2.27	1.47	1.19	2.13	1.75(1.74-1.78)	1.064	
	mRFP	Trehalose	0.0 (M)	3.21	1.50	0.97	2.29	1.75(1.74-1.77)	1.080	
	mRFP	Trehalose	0.2 (M)	0.55	0.74	5.74	1.74	1.70(1.69-1.70)	1.131	
	mRFP	Trehalose	0.4 (M)	1.97	1.47	0.72	2.19	1.72(1.71-1.76)	1.067	
	mRFP	Trehalose	0.7 (M)	1.35	1.30	1.31	1.97	1.70(1.67-1.72)	1.018	
	mRFP	Trehalose	1.0 (M)	2.17	1.84	1.45	1.16	1.63(1.63-1.64)	1.142	
	mRFP	Trehalose	1.3 (M)	0.83	0.74	4.99	1.67	1.60(1.60-1.61)	1.131	
	mRFP	Glycerol	0 (%)	3.21	1.50	0.97	2.29	1.75(1.74-1.77)	1.080	
	mRFP	Glycerol	5 (%)	1.76	1.58	0.24	2.70	1.79(1.77-1.87)	1.056	
	mRFP	Glycerol	10 (%)	2.04	1.50	0.84	2.17	1.75(1.74-1.78)	1.028	
	mRFP	Glycerol	12 (%)	2.11	1.59	0.15	3.04	1.76(1.74-1.79)	1.078	
	mRFP	Glycerol	14 (%)	0.50	0.94	2.85	1.79	1.72(1.71-1.73)	1.005	
	mRFP	Glycerol	16 (%)	1.96	1.59	0.11	3.30	1.77(1.73-1.80)	1.058	
	mRFP	Glycerol	20 (%)	0.46	2.37	2.00	1.53	1.75(1.74-1.78)	1.132	
	mRFP	Glycerol	25 (%)	0.59	2.38	2.38	1.52	1.76(1.74-1.80)	1.149	
	mRFP	Glycerol	30 (%)	1.09	1.26	1.58	1.93	1.72(1.71-1.74)	1.073	
	mRFP	Glycerol	40 (%)	0.80	0.80	4.58	1.74	1.67(1.67-1.67)	1.044	
	mRFP	Glycerol	50 (%)	0.67	0.95	2.57	1.78	1.68(1.67-1.68)	1.019	
	mRFP	PEG 3,350	0 (% FVO)	3.21	1.50	0.97	2.29	1.75(1.74-1.77)	1.080	
	mRFP	PEG 3,350	2 (% FVO)	0.55	0.94	3.67	1.80	1.74(1.74-1.75)	1.038	
	mRFP	PEG 3,350	7 (% FVO)	3.14	1.85	0.80	1.10	1.75(1.74-1.76)	1.015	
	mRFP	PEG 3,350	15 (% FVO)	0.58	0.77	6.56	1.76	1.72(1.72-1.73)	1.280	
	mRFP	PEG 3,350	20 (% FVO)	0.75	0.91	3.89	1.78	1.70(1.70-1.71)	1.048	
	mRFP	PEG 3,350	30 (% FVO)	0.92	0.78	6.54	1.76	1.70(1.70-1.70)	1.133	
	mRFP	PEG 3,350	40 (% FVO)	1.34	0.91	4.66	1.80	1.69(1.69-1.69)	1.160	
	mRFP	PEG 3,350	50 (% FVO)	2.76	0.59	1.78	1.75	1.35(1.34-1.36)	1.262	
	mRFP	PEG 6,000	0 (% FVO)	3.21	1.50	0.97	2.29	1.75(1.74-1.77)	1.080	
	mRFP	PEG 6,000	2 (% FVO)	0.67	0.86	7.00	1.77	1.73(1.73-1.74)	1.254	
	mRFP	PEG 6,000	7 (% FVO)	1.18	1.23	2.71	1.88	1.74(1.73-1.76)	1.028	
	mRFP	PEG 6,000	15 (% FVO)	0.90	0.94	6.86	1.77	1.72(1.71-1.72)	1.272	
	mRFP	PEG 6,000	20 (% FVO)	1.07	0.88	4.90	1.80	1.71(1.71-1.72)	1.004	
	mRFP	PEG 6,000	30 (% FVO)	6.13	1.77	1.22	0.77	1.69(1.69-1.70)	1.058	
	mRFP	PEG 6,000	40 (% FVO)	1.57	1.87	1.43	0.86	1.57(1.57-1.58)	1.145	
	mRFP	PEG 6,000	50 (% FVO)	0.32	1.75	1.87	0.58	0.98(0.96-1.00)	1.408	
	mRFP	PEG 10,000	0 (% FVO)	3.21	1.50	0.97	2.29	1.75(1.74-1.77)	1.080	
	mRFP	PEG 10,000	2 (% FVO)	1.45	1.38	1.29	2.03	1.75(1.74-1.78)	1.060	
	mRFP	PEG 10,000	7 (% FVO)	2.97	1.87	1.02	1.17	1.74(1.74-1.76)	1.039	
	mRFP	PEG 10,000	15 (% FVO)	0.58	0.80	4.63	1.76	1.71(1.71-1.72)	1.106	
	mRFP	PEG 10,000	20 (% FVO)	0.82	0.86	4.79	1.78	1.70(1.70-1.71)	1.078	
	mRFP	PEG 10,000	30 (% FVO)	1.60	0.93	3.93	1.84	1.68(1.68-1.69)	1.033	
	mRFP	PEG 10,000	40 (% FVO)	2.84	0.81	2.28	1.81	1.45(1.45-1.46)	1.124	
	mRFP	PEG 10,000	50 (% FVO)	2.78	0.53	0.25	1.65	0.78(0.76-0.80)	1.319	
	eGFP	NaCl	0.0 (M)	1.36	2.40	0.23	3.65	2.66 (2.65-2.67)	1.296	
	eGFP	NaCl	0.05 (M)	0.21	1.67	1.02	2.75	2.64 (2.60-2.65)	1.132	
	eGFP	NaCl	0.1 (M)	1.56	2.67	0.11	1.38	2.62 (2.62-2.63)	1.469	
	eGFP	NaCl	0.2 (M)	0.07	4.13	0.65	2.45	2.68 (2.65-2.68)	1.132	
	eGFP	NaCl	0.4 (M)	0.65	2.39	0.13	3.58	2.66 (2.65-2.67)	1.136	
	eGFP	NaCl	0.7 (M)	0.55	2.47	0.02	5.33	2.70 (2.67-2.72)	1.094	
	eGFP	NaCl	1.0 (M)	0.59	2.40	0.07	3.89	2.65 (2.64-2.66)	1.113	
	eGFP	NaCl	1.5 (M)	0.69	2.36	0.14	3.44	2.61 (2.60-2.62)	1.187	
	eGFP	NaCl	2.0 (M)	0.59	2.36	0.04	4.38	2.62 (2.60-2.62)	1.033	
	eGFP	Trehalose	0.0 (M)	1.36	2.40	0.23	3.65	2.66 (2.65-2.67)	1.296	
	eGFP	Trehalose	0.2 (M)	0.48	2.05	0.63	2.92	2.62 (2.61-2.64)	1.005	
	eGFP	Trehalose	0.4 (M)	0.16	3.57	0.80	2.32	2.61 (2.60-2.65)	1.075	
	eGFP	Trehalose	0.7 (M)	0.68	2.18	0.26	3.17	2.53 (2.51-2.55)	1.042	
	eGFP	Trehalose	1.0 (M)	0.38	1.56	0.87	2.63	2.41 (2.41-2.42)	1.070	
	eGFP	Trehalose	1.3 (M)	0.38	1.33	1.6	2.48	2.35 (2.35-2.35)	1.029	
	eGFP	Glycerol	0 (%)	1.36	2.40	0.23	3.65	2.66 (2.65-2.67)	1.296	
	eGFP	Glycerol	5 (%)	1.05	2.72	0.18	1.68	2.61 (2.61-2.62)	1.142	
	eGFP	Glycerol	10 (%)	1.70	2.61	0.09	1.22	2.57 (2.57-2.57)	1.333	
	eGFP	Glycerol	12 (%)	1.77	2.60	0.11	1.24	2.56 (2.56-2.57)	1.394	
	eGFP	Glycerol	14 (%)	1.13	2.65	0.18	1.59	2.56 (2.55-2.56)	1.084	
	eGFP	Glycerol	16 (%)	2.10	2.58	0.13	1.22	2.54 (2.54-2.55)	1.445	
	eGFP	Glycerol	20 (%)	2.37	2.54	0.13	0.93	2.50 (2.50-2.51)	1.574	
	eGFP	Glycerol	25 (%)	2.18	2.51	0.16	0.92	2.47 (2.47-2.48)	1.344	
	eGFP	Glycerol	30 (%)	2.43	2.48	0.15	0.84	2.45 (2.45-2.45)	1.505	
	eGFP	Glycerol	40 (%)	2.09	2.44	0.15	0.97	2.40 (2.40-2.41)	1.239	
	eGFP	Glycerol	50 (%)	1.93	2.43	0.24	1.15	2.36 (2.36-2.37)	1.095	
	eGFP	PEG 3,350	0 (% FVO)	1.36	2.40	0.23	3.65	2.66 (2.65-2.67)	1.296	
	eGFP	PEG 3,350	2 (% FVO)	0.56	2.34	0.15	3.50	2.67 (2.65-2.70)	1.063	
	eGFP	PEG 3,350	7 (% FVO)	0.15	1.13	1.53	2.59	2.53 (2.53-2.54)	1.096	
	eGFP	PEG 3,350	15 (% FVO)	0.05	1.70	0.19	2.72	2.57 (2.55-2.61)	1.020	
	eGFP	PEG 3,350	20 (% FVO)	0.13	1.14	1.79	2.55	2.51 (2.50-2.51)	1.222	
	eGFP	PEG 3,350	30 (% FVO)	0.18	0.86	2.85	2.47	2.44 (2.43-2.44)	1.523	
	eGFP	PEG 3,350	40 (% FVO)	0.34	0.93	2.52	2.28	2.21 (2.20-2.21)	1.525	
	eGFP	PEG 3,350	50 (% FVO)	0.32	0.78	3.49	2.31	2.27 (2.26-2.27)	1.606	

eGFP	PEG 6,000	0 (% FVO)	1.36	2.40	0.23	3.65	2.66 (2.65-2.67)	1.296
eGFP	PEG 6,000	2 (% FVO)	0.69	2.31	0.26	3.30	2.66 (2.65-2.67)	1.059
eGFP	PEG 6,000	7 (% FVO)	0.30	1.52	2.22	2.69	2.61 (2.60-2.61)	1.131
eGFP	PEG 6,000	15 (% FVO)	0.18	0.99	3.30	2.58	2.55 (2.55-2.55)	1.437
eGFP	PEG 6,000	20 (% FVO)	0.26	0.75	4.40	2.54	2.50 (2.50-2.51)	1.640
eGFP	PEG 6,000	30 (% FVO)	0.28	0.80	4.21	2.45	2.42 (2.41-2.42)	1.635
eGFP	PEG 6,000	40 (% FVO)	0.31	0.88	3.30	2.40	2.35 (2.35-2.35)	1.393
eGFP	PEG 6,000	50 (% FVO)	0.33	1.03	1.38	2.24	2.12 (2.11-2.12)	1.252
eGFP	PEG 10,000	0 (% FVO)	1.36	2.40	0.23	3.65	2.66 (2.65-2.67)	1.296
eGFP	PEG 10,000	2 (% FVO)	0.13	1.20	1.99	2.65	2.61 (2.61-2.61)	1.302
eGFP	PEG 10,000	7 (% FVO)	2.73	2.61	0.13	0.91	2.58 (2.58-2.62)	1.381
eGFP	PEG 10,000	15 (% FVO)	3.93	2.57	0.22	0.76	2.54 (2.54-2.55)	1.656
eGFP	PEG 10,000	20 (% FVO)	3.94	2.52	0.25	0.65	2.49 (2.49-2.50)	1.729
eGFP	PEG 10,000	30 (% FVO)	3.10	2.48	0.23	0.56	2.45 (2.44-2.45)	1.659
eGFP	PEG 10,000	40 (% FVO)	3.26	2.42	0.26	0.85	2.38 (2.37-2.39)	1.517
eGFP	PEG 10,000	50 (% FVO)	0.31	0.99	1.98	2.27	2.19 (2.19-2.19)	1.282
mCitrine	NaCl	0.0 (M)	0.25	3.32	0.74	3.33	3.32(3.32-3.35)	1.101
mCitrine	NaCl	0.1 (M)	0.01	0.01	1.07	3.35	3.34(3.35-3.45)	1.263
mCitrine	NaCl	0.2 (M)	0.21	2.65	0.94	3.44	3.32(3.16-3.34)	1.060
mCitrine	NaCl	0.4 (M)	0.02	0.01	1.12	3.32	3.32(3.31-3.32)	1.167
mCitrine	NaCl	0.7 (M)	0.10	2.20	1.12	3.35	3.29(3.20-3.30)	1.084
mCitrine	NaCl	1.0 (M)	0.01	0.01	1.31	3.25	3.25(3.23-3.25)	1.108
mCitrine	NaCl	1.5 (M)	0.15	1.81	1.02	3.32	3.21(2.97-3.26)	1.078
mCitrine	NaCl	2.0 (M)	0.19	2.02	0.87	3.27	3.12(2.99-3.18)	1.178
mCitrine	Trehalose	0.0 (M)	0.25	3.32	0.74	3.33	3.32(3.32-3.35)	1.101
mCitrine	Trehalose	0.2 (M)	0.01	0.01	1.04	3.28	3.28(3.25-3.32)	1.048
mCitrine	Trehalose	0.4 (M)	0.11	3.24	1.00	3.24	3.24(3.24-3.37)	1.114
mCitrine	Trehalose	0.7 (M)	0.04	2.40	1.11	3.23	3.21(3.21-3.31)	1.105
mCitrine	Trehalose	1.0 (M)	0.36	2.80	1.20	3.16	3.08(3.08-3.17)	1.108
mCitrine	Trehalose	1.3 (M)	0.94	3.06	0.01	3.03	3.06(3.06-3.24)	1.107
mCitrine	Glycerol	0 (%)	0.25	3.32	0.74	3.33	3.32(3.32-3.35)	1.101
mCitrine	Glycerol	5 (%)	0.01	9.43	1.08	3.07	3.32(3.25-3.38)	1.060
mCitrine	Glycerol	10 (%)	0.01	9.68	1.11	3.05	3.28(3.21-3.51)	1.050
mCitrine	Glycerol	12 (%)	-	-	-	-	-	-
mCitrine	Glycerol	14 (%)	-	-	-	-	-	-
mCitrine	Glycerol	16 (%)	0.02	7.34	1.19	2.99	3.14(3.11-3.15)	1.099
mCitrine	Glycerol	20 (%)	0.01	9.63	1.24	2.95	3.17(3.11-3.19)	1.090
mCitrine	Glycerol	25 (%)	0.01	11.86	1.13	2.94	3.17(3.11-3.17)	1.016
mCitrine	Glycerol	30 (%)	-	-	-	-	-	-
mCitrine	Glycerol	40 (%)	0.07	4.56	1.80	2.80	2.90(2.87-2.99)	1.144
mCitrine	Glycerol	50 (%)	0.01	9.47	1.55	2.83	2.90(2.85-2.91)	1.185
mCitrine	PEG 3,350	0 (% FVO)	0.25	3.32	0.74	3.33	3.32(3.32-3.35)	1.101
mCitrine	PEG 3,350	2 (% FVO)	0.36	3.39	-	-	3.39(3.35-3.40)	1.041
mCitrine	PEG 3,350	7 (% FVO)	0.10	2.22	0.77	3.37	3.28(3.27-3.29)	1.062
mCitrine	PEG 3,350	15 (% FVO)	-	-	-	-	-	-
mCitrine	PEG 3,350	20 (% FVO)	0.03	1.34	0.91	3.24	3.21(3.20-3.22)	1.009
mCitrine	PEG 3,350	30 (% FVO)	0.05	1.63	0.98	3.14	3.10(3.09-3.10)	1.063
mCitrine	PEG 3,350	40 (% FVO)	0.29	3.36	0.77	2.25	2.65(2.60-2.68)	1.125
mCitrine	PEG 3,350	50 (% FVO)	0.91	2.20	0.19	3.27	2.45(2.41-2.53)	1.120
mCitrine	PEG 6,000	0 (% FVO)	0.25	3.32	0.74	3.33	3.32(3.32-3.35)	1.101
mCitrine	PEG 6,000	2 (% FVO)	0.00	0.01	0.88	3.33	3.33(3.22-3.33)	1.172
mCitrine	PEG 6,000	7 (% FVO)	0.01	3.32	0.80	3.32	3.32(3.32-3.32)	1.171
mCitrine	PEG 6,000	15 (% FVO)	0.19	3.23	0.86	3.23	3.23(3.23-3.45)	1.128
mCitrine	PEG 6,000	20 (% FVO)	-	-	-	-	-	-
mCitrine	PEG 6,000	30 (% FVO)	0.76	2.59	0.17	3.74	2.87(2.86-2.90)	1.072
mCitrine	PEG 6,000	40 (% FVO)	0.61	2.54	0.01	7.54	2.79(2.74-2.89)	1.072
mCitrine	PEG 6,000	50 (% FVO)	0.01	0.01	1.47	2.46	2.46(2.45-2.52)	1.149
mCitrine	PEG 10,000	0 (% FVO)	0.25	3.32	0.74	3.33	3.32(3.32-3.35)	1.101
mCitrine	PEG 10,000	2 (% FVO)	0.05	2.01	0.81	3.40	3.35(3.34-3.36)	1.019
mCitrine	PEG 10,000	7 (% FVO)	0.68	3.17	0.04	4.93	3.32(3.30-3.35)	1.055
mCitrine	PEG 10,000	15 (% FVO)	0.36	3.22	1.18	3.22	3.22(3.20-3.22)	1.197
mCitrine	PEG 10,000	20 (% FVO)	-	-	-	-	-	-
mCitrine	PEG 10,000	30 (% FVO)	0.66	2.30	0.38	3.37	2.79(2.78-2.80)	1.011
mCitrine	PEG 10,000	40 (% FVO)	0.92	2.72	0.48	1.97	2.51(2.36-2.54)	1.145
mCitrine	PEG 10,000	50 (% FVO)	1.17	2.62	0.75	1.15	2.29(2.15-2.70)	1.111

Supplementary Table 2: a list of the residues exhibiting a network of contacts with the fluorophore of mCherry in the MD simulations

Residue	Probability (%) at 0% FVO PEG	Probability (%) at 42% FVO PEG
F14	84.7	77.6
Q42	99.9	99.5
T43	88.3	75.7
A44	99.8	99.5
L46	6.3	8.7
S62	16.8	24.6
P63	99.9	100.0
Q64	87.2	84.9
F65	100.0	100.0
S69	100.0	100.0
K70	65.7	63.4
F91	3.1	4.3
W93	99.3	98.6
R95	100.0	100.0
Q109	49.9	51.6
W143	31.7	29.0
E144	2.4	1.5
A145	15.3	17.2
S146	41.1	47.8
E148	4.2	7.1
I161	95.4	99.4
Q163	85.7	83.0
V177	0.7	1.4
Y181	29.0	37.4
I197	99.5	99.6
K198	3.7	1.7
L199	100.0	100.0
Q213	99.7	99.8
Y214	57.1	49.2
E215	100.0	100.0
