# Supplementary material for: Fluorescent protein lifetimes report densities and phases of nuclear condensates during embryonic stem-cell differentiation
Source: Nat Commun. 2023 Aug 12;14:4885. doi: 10.1038/s41467-023-40647-6 (PMC10423231; doi:10.1038/s41467-023-40647-6)
Supplement: Supplementary file 3 — Description of Additional Supplementary Files [file 41467_2023_40647_MOESM3_ESM.pdf]

Description of Additional Supplementary Files

Supplementary Movie 1: Lifetimes of PEG-induced in vitro mCherry precipitates in solution over time, in 25% PEG FVO.

Supplementary Movie 2: Lifetimes of PEG-induced in vitro mCherry precipitates in solution over time, in 33% PEG FVO.
