# Supplementary material for: Fluorescent protein lifetimes report densities and phases of nuclear condensates during embryonic stem-cell differentiation
Source: Nat Commun. 2023 Aug 12;14:4885. doi: 10.1038/s41467-023-40647-6 (PMC10423231; doi:10.1038/s41467-023-40647-6)
Supplement: Supplementary file 6 — Reporting Summary [file 41467_2023_40647_MOESM6_ESM.pdf]

nature portfolio

Corresponding author(s): Eran Meshorer, Eitan Lerner

Last updated by author(s): Jul 20, 2023

Reporting Summary

Nature Portfolio wishes to improve the reproducibility of the work that we publish. This form provides structure for consistency and transparency in reporting. For further information on Nature Portfolio policies, see our Editorial Policies and the Editorial Policy Checklist.

Statistics

For all statistical analyses, confirm that the following items are present in the figure legend, table legend, main text, or Methods section.

n/a	Confirmed	
☐	☒	The exact sample size (*n*) for each experimental group/condition, given as a discrete number and unit of measurement
☐	☒	A statement on whether measurements were taken from distinct samples or whether the same sample was measured repeatedly
☐	☒	The statistical test(s) used AND whether they are one- or two-sided<br>*Only common tests should be described solely by name; describe more complex techniques in the Methods section.*
☒	☐	A description of all covariates tested
☒	☐	A description of any assumptions or corrections, such as tests of normality and adjustment for multiple comparisons
☐	☒	A full description of the statistical parameters including central tendency (e.g. means) or other basic estimates (e.g. regression coefficient) AND variation (e.g. standard deviation) or associated estimates of uncertainty (e.g. confidence intervals)
☐	☒	For null hypothesis testing, the test statistic (e.g. *F*, *t*, *r*) with confidence intervals, effect sizes, degrees of freedom and *P* value noted<br>*Give P values as exact values whenever suitable.*
☒	☐	For Bayesian analysis, information on the choice of priors and Markov chain Monte Carlo settings
☒	☐	For hierarchical and complex designs, identification of the appropriate level for tests and full reporting of outcomes
☒	☐	Estimates of effect sizes (e.g. Cohen's *d*, Pearson's *r*), indicating how they were calculated

Our web collection on statistics for biologists contains articles on many of the points above.

Software and code

Policy information about availability of computer code

Data collection	ISS Vistavision software (version 4.2.095) was used to track the raw data collection for in vitro fluorescence decays and in cell FLIM and FCS measurements
Data analysis	Retrieving the in vitro mean fluorescence lifetime values from best-fit results of fitting sum-of-exponentials function to fluorescence decays using a Fortran-based software from Haas and colleagues. Details and references are provided in the manuscript. References are also provided in Beechem & Haas. Biophysical Journal (1989) and in Huang, Lerner et al. Biochemistry (2009).<br>The analyses of pixel-wise fluorescence lifetimes, mean fluorescence lifetimes and FCS curves were performed in ISS VistaVision software (version 4.2.095). The analyses of photobleaching curves were performed using OriginLab Origin software (version 2022b).<br>The initial structure for MD simulations was prepared with CHIMERA (version 1.15).<br>MD simulation data was collected and analyzed using GROMACS (version 2022.2), Python (version 3.7), the LINCS and SETTLE algorithms, as well as the MDAnalysis module (version 2.2.0). The convex hull was computed with the Open3D module (version 0.15.0).

For manuscripts utilizing custom algorithms or software that are central to the research but not yet described in published literature, software must be made available to editors and reviewers. We strongly encourage code deposition in a community repository (e.g. GitHub). See the Nature Portfolio guidelines for submitting code & software for further information.

Data

Policy information about availability of data

All manuscripts must include a data availability statement. This statement should provide the following information, where applicable:

- Accession codes, unique identifiers, or web links for publicly available datasets
- A description of any restrictions on data availability
- For clinical datasets or third party data, please ensure that the statement adheres to our policy

The raw fluorescence spectra, decays, and anisotropy decays, as well as the fluorescence decay fitting results generated in this study have been deposited in a publicly available Zenodo repository [https://doi.org/10.5281/zenodo.7964824]. This repository also includes the raw in-cell fluorescence intensity trajectories, their autocorrelation curves and their analyses, as well as the raw FLIM data and their analyses. Finally, this repository also includes the full MD simulation trajectory and its analyses results. Description of the different data provided in the repository is found in the Source Data file. The crystal structure of mCherry serving as the bases for MD simulations was taken from the Protein Data Bank (PDB ID 2H5Q).

Human research participants

Policy information about studies involving human research participants and Sex and Gender in Research.

Reporting on sex and gender	n/a
Population characteristics	n/a
Recruitment	n/a
Ethics oversight	n/a

Note that full information on the approval of the study protocol must also be provided in the manuscript.

Field-specific reporting

Please select the one below that is the best fit for your research. If you are not sure, read the appropriate sections before making your selection.

☒ Life sciences ☐ Behavioural & social sciences ☐ Ecological, evolutionary & environmental sciences

For a reference copy of the document with all sections, see nature.com/documents/nr-reporting-summary-flat.pdf

Life sciences study design

All studies must disclose on these points even when the disclosure is negative.

Sample size	n/a
Data exclusions	n/a
Replication	All attempts of replication were successful.
Randomization	n/a
Blinding	n/a

Reporting for specific materials, systems and methods

We require information from authors about some types of materials, experimental systems and methods used in many studies. Here, indicate whether each material, system or method listed is relevant to your study. If you are not sure if a list item applies to your research, read the appropriate section before selecting a response.

Materials & experimental systems

n/a	Involved in the study
☒	☐ Antibodies
☐	☒ Eukaryotic cell lines
☒	☐ Palaeontology and archaeology
☒	☐ Animals and other organisms
☒	☐ Clinical data
☒	☐ Dual use research of concern

Methods

n/a	Involved in the study
☒	☐ ChIP-seq
☒	☐ Flow cytometry
☒	☐ MRI-based neuroimaging

Eukaryotic cell lines

Policy information about cell lines and Sex and Gender in Research

Cell line source(s)	R1 mouse embryonic stem cells (ATCC)
Authentication	Cells were previously authenticated (Harikumar, et al 2017)
Mycoplasma contamination	Cell line was tested for mycoplasma with MycoBlue Mycoplasma Detector kit from Vazyme, Nanjing, China (D101-02)
Commonly misidentified lines (See ICLAC register)	none
